# *Madhuca longifolia* bark extract as an adjunct to the treatment of colon carcinoma cells with SN-38: a synergistic combination with potent activity

**DOI:** 10.3389/fphar.2026.1804862

**Published:** 2026-05-12

**Authors:** Anna Barycka, Kamila Środa-Pomianek, Michał Gleńsk, Meena Rajbhandari, Magdalena Skonieczna, Anna Palko-Łabuz, Olga Wesołowska

**Affiliations:** 1 Department of Biophysics and Neuroscience, Wroclaw Medical University, Wrocław, Poland; 2 Department of Pharmacognosy and Herbal Medicines, Wrocław Medical University, Wrocław, Poland; 3 Research Centre for Applied Science and Technology (RECAST), Kathmandu, Nepal; 4 Department of Systems Biology and Engineering, The Silesian University of Technology, Gliwice, Poland; 5 Biotechnology Centre, Silesian University of Technology, Gliwice, Poland

**Keywords:** bark, colon cancer, cytotoxicity, extract, irinotecan, *Madhuca longifolia*, synergy

## Abstract

**Background:**

Plants have been used by humans as a source of food, spices, and medicines since prehistoric times. *Madhuca longifolia* is a tree native to South Asia. In traditional medicine of the region, extracts from different parts of this plant are used to treat inflammation, ulcers, epilepsy, diabetes, and rheumatism.

**Methods:**

Cytotoxicity of the extract from the bark of *M. longifolia* and its fractions was assessed via sulforhodamine B (SRB) assay in human colorectal cancer cell lines HT-29 and HCT116, as well as normal epithelial cells FHC. The effects on cell cycle progression, apoptosis induction, and reactive oxygen species (ROS) production were studied by flow cytometry. The existence of synergy between the extract and active form of irinotecan, SN-38, was investigated by means of CompuSyn software.

**Results:**

Aqueous ethanolic extract from *M. longifolia* was demonstrated to exhibit potent cytotoxic effect in human colon cancer cells. The studied extract and its fractions exhibited some degree of selectivity, since they were more toxic to cancer cells than to normal colon epithelium. Additionally, the extract and its fractions turned out to potentiate anticancer effect of SN-38 used in concentrations that were non-toxic to the cells. The most active extract was shown to induce apoptosis and increase ROS production in cancer cells. The anticancer effect of the combination of SN-38 and *M. longifolia* extract increased even further when the extract was applied to the cells a few hours before the drug. These results indicate that *M. longifolia* bark extract sensitized colon cancer cells to SN-38.

**Conclusion:**

These findings provide pharmacological support for the traditional use of *M. longifolia* and suggest that its bark extract may constitute a source of promising adjunct substances that could be potentially used in therapy in order to decrease the concentrations of irinotecan applied to the patients and, in this way, reduce side-effects of the drug.

## 1 Introduction

Plants have been used by humans as a source of food, spices, and medicines since prehistoric times. Based on the data from China, where nearly 19% of plant species are used as drugs in traditional medicine (Duke and Ayensu, 1985), it can be estimated that the total number of medicinal plant species worldwide reaches approximately 50,000 (Schippmann et al., 2002). Evidence of medicinal use of plants date back to ancient Sumer (c.a. 5,000 years ago), through ancient civilizations of Egypt, China, India, and the Mediterranean (Halberstein, 2005). According to WHO, even nowadays about 80% of people in developing countries predominantly use traditional botanical drugs in treatment of various ailments (WHO, 2013) For years, plants have constituted the source of biologically active substances, and it is estimated that more than 25% of modern drugs is of natural origin (Thomford et al., 2018; Newman and Cragg, 2020).

*Madhuca longifolia* is a fast-growing, evergreen tree native to India, Nepal, Myanmar, and Sri Lanka. It is cultivated for its oily seeds, edible flowers and fruits. In the traditional medicine of the region, extracts from different parts of this plant are used to treat inflammation, ulcers, epilepsy, diabetes, and rheumatism (Yadav et al., 2012; Sinha et al., 2017). Significant antioxidant, antibacterial, anticancer, as well as anti-inflammatory properties of *M. longlifolia* extracts have been demonstrated (Shaban et al., 2012; Jha and Mazumder, 2018; Jose et al., 2018; Dubey et al., 2019). The phytochemical analysis of different parts *M. longlifolia* plants revealed many compounds of biological activity, including flavonoids and their glycosydes, tannins, steroids, saponins, and triterpenoids (Chinna Eswaraiah et al., 2011; Gopalkrishnan and Shimpi, 2012; Bürkel et al., 2023).

Plants contain thousands of bioactive compounds, most of which are their secondary metabolites. Among the most important classes of compounds of potent biological activity alkaloids, polyphenols, glycosides, and terpenoids can be listed. Multi-component composition of plant extracts frequently results in the modulation of the activity of individual components due to the interactions between them. These interactions might be additive, antagonistic, or synergistic, i.e., the activity of the mixture is higher than the sum of activities of individual components. The last type of interaction is of particular interest from the pharmacological point of view. Knowledge of the properties of medicinal plants gathered over generations led traditional healers to use combinations of various plant products in their recipes.

Such an approach is close to the modern way of therapy of diseases such as cancer, HIV, and cardiovascular diseases in which drug combinations are preferentially used. It is based on the assumption that multifactorial diseases require a treatment that could affect many targets simultaneously. Multidrug regiments have been proven to control complex diseases better, and are believed to be less susceptible to drug resistance (Zimmermann et al., 2007).

Colorectal carcinoma occupies nowadays the third position among the most frequently occurring cancers in the world (WHO, 2026). Chemotherapy is widely used in the treatment of this type of cancer, especially when the disease is metastatic (Kanemitsu et al., 2021). Commonly used drug combinations include fluorouracil, leucovorin, and irinotecan (FOLFIRI) or fluorouracil, leucovorin, oxaliplatin, and irinotecan (FOLFOXIRI) (van Cutsem et al., 2016; Yoshino et al., 2018). Irinotecan was derived from a naturally occurring alkaloid, camptothecin. Both are inhibitors of topoisomerase I (Rivory, 1996). The active form of irinotecan, SN-38, is formed by the hydrolysis of the prodrug by patients’ carboxylesterases. Diarrhea, nausea, and vomiting are the most common side effects of irinotecan that result from the toxicity of the drug to intestinal epithelium (Gibson and Stringer, 2009; Stein et al., 2010).

In the present work, anticancer properties of the extracts from the bark of *M. longifolia* have been investigated in two colon cancer cell lines. Our previous study has shown high activity of one *M. longifolia* extract in human melanoma cells (Środa-Pomianek et al., 2024). This study provides the first evidence that bark extracts of *M. longifolia* sensitize colorectal cancer cells to the active metabolite of irinotecan, SN-38. It was demonstrated that the extract exhibited selective cytotoxicity toward cancer cells, induced apoptosis and reactive oxygen species production, and significantly enhanced the anticancer activity if both the extract and SN-38 were applied simultaneously. Importantly, pre-treatment with the extract further strengthened this synergistic effect. These findings reveal a previously unreported chemosensitizing potential of *M. longifolia* bark extract and highlight its promise as a natural adjuvant that may allow reduction of irinotecan doses and associated side effects in colorectal cancer therapy.

## 2 Materials and methods

### 2.1 Chemicals

Camptothecin and the active form of irinothecan, SN-38, were obtained from Sigma Aldrich (Poznań, Poland). Both drugs were dissolved in dimethyl sulfoxide (DMSO) and stored at −20 °C. Sulforhodamine B (SRB), MTT (3-(4,5-dimethylthiazol-2-yl)-2,5-diphenyltetrazolium bromide), sodium dodecyl sulphate (SDS), hydrogen peroxide, propidium iodide (PI), 2′,7′-dichlorofluorescin diacetate (DCFH-DA), N-acetylcysteine (NAC), and rhodamine123 (R123) were purchased from Sigma Aldrich (Poznań, Poland).

### 2.2 Plant material

The bark of *M. longifolia* (J. Koenig ex L.) J. F. Macbr. was collected in August 2016 at Kailali district of western Nepal. For our research purpose, the barks were collected from the tree growing in the private lands with the permission of landowners as wasted product during debarking process. The plant was authenticated by Prof. Dr. Sangeeta Rajbhandary, Central Department of Botany, Tribhuvan University. A voucher specimen (BL-16-DPP) was deposited at the Research Centre for Applied Science and Technology (RECAST), Tribhuvan University, Kathmandu, Nepal. The collected materials were washed, the outer layers of the bark was scrapped and it was cut into small pieces and dried away from sunlight. The dried materials were properly packed and sent to Poland with the permission of Department of Plant Resources, Ministry of Forest and Environment, Government of Nepal through General post office, Government of Nepal. It has been also deposited (ML_01) in the Herbarium of Department of Pharmacognosy and Herbal Medicines, Wroclaw Medical University, Wroclaw, Poland.

### 2.3 Extraction

The dried bark of *M. longifolia* was grounded into powder using a mill (Ika A11, Staufen, Germany) and macerated with 70% aqueous ethanol at room temperature for 24 h. The drug-to-solvent ratio was 1–10 (g/mL). After filtration, the extract was evaporated on a rotavapor R-210 (Buchi, Flavil, Switzerland) and the sticky residue was dried in a vacuum chamber for the next 24 h, giving the final MLE extract. Next, the part of MLE extract (200 mg) was suspended in water and then partitioned with dichloromethane and ethyl acetate, respectively. As a result of partitioning, three fractions were collected: MLDCM–dichloromethane fraction (10 mg); MLOA–ethyl acetate fraction (90 mg), and MLWR water fraction (100 mg). All of them were dried and analyzed by UHPLC-ESI-MS.

### 2.4 Analysis

The UHPLC-ESI-MS analyses were performed according to the method described previously (Środa-Pomianek et al., 2024).

### 2.5 Cell culture

Human colorectal cancer cell lines HT-29 and HCT116 as well as normal epithelial cells FHC were purchased from ATCC collection (Manassas, United States). Madin-Darby Canine Kidney cells (MDCK) and MDCK cells expressing human ABCB1 (MDCK-MDR1) (Pastan et al., 1988) were purchased from the Netherlands Cancer Institute (NKI-AVL, Amsterdam, Netherlands). Cell culture medium DMEM-F12 (Cytogen Zgierz, Poland) was used, and supplemented with 1% Penicillin-Streptomycin (Sigma Aldrich, Poznań, Poland), 1% L-glutamine (Sigma Aldrich, Poznań, Poland), and 10% of fetal bovine serum (FBS, Gibco, Waltham, United States). Medium for FHC cells was additionally supplemented with 0.005 mg/mL insulin, 0.005 mg/mL transferrin, 100 ng/mL hydrocortisone, 20 ng/mL human recombinant EGF, and 10 ng/mL cholera toxin. Cells were cultivated in a humidified atmosphere (37 °C, 5% CO_2_).

### 2.6 Cytotoxicity assay

Determination of cytotoxicity of the studied extracts was performed by means of the sulforhodamine B (SRB) assay (Skehan et al., 1990), and cell viability was measured by MTT (3-(4,5-dimethylthiazol-2-yl)-2,5-diphenyltetrazolium bromide) assay. MTT assay is based on the reduction of a yellow tetrazolium salt to purple formazan crystals by metabolically active cells. Colon cells were seeded (60,000/well) onto 96-well plates with the studied substances in the appropriate concentrations. Concentration of DMSO in the samples did not exceed 1%, and the influence of the solvent itself on the cells was monitored. The plates were incubated for 48 h at 37 °C. SRB assay was stopped by the addition of 50% cold trichloroacetic acid and incubation for 60 min at 4 °C. Then the plates were washed with tap water and stained with 0.02% sulforhodamine B (Sigma-Aldrich, Poznan, Poland) for 60 min at room temperature. The excess of the dye was removed by washing with 1% acetic acid, and the plates were dried. Next, 10 mmol/L Tris (pH = 10.5) was added to each well. Absorbance at 491 nm was measured to estimate protein concentration in the samples. Survival rate was calculated as the ratio of A_491_ of treated cells by A_491_ of control cells times 100%. MTT assay was finished by removal of the medium from the plates and addition 0.5 mg/mL of MTT followed by incubation for 4 h at 37 °C. Formazan crystals were then dissolved in isopropanol:HCl mixture (v/v 1:0.04) and the absorbance was read at 570 nm. Cell viability was calculated as the ratio of A_570_ of treated cells by A_570_ of control cells times 100%. In both assays SDS was used as a positive control (Supplementary Figures S1, S3).

### 2.7 Drug-extract interaction analysis

For the analysis of the mode of interaction between anticancer drug and *M. longifolia* extracts CompuSyn software (ComboSyn Inc., Paramus, United States) was applied. The combination index (CI) was calculated according to the classic median-effect Equation 1, as was previously described (Chou and Martin, 2005).

$$CI=\frac{{\left(D\right)}_{1}}{{\left(Dx\right)}_{1}}+\frac{{\left(D\right)}_{2}}{{\left(Dx\right)}_{2}}$$

In the equation (Dx)_1_ is the dose of drug 1 alone that inhibits a system by x%, (Dx)_2_ is the dose of drug 2 alone that inhibits a system by x%, and (D)_1_ + (D)_2_ are the doses of drug 1 and 2 in combination that also inhibit a system by x%. If CI values are below 1, they point to synergistic interaction; if CI value are equal to 1, they indicate an additive effect, and if CI values are above 1, they suggest antagonism between the studied drugs.

### 2.8 Flow cytometry

Cell cycle, apoptosis, and intracellular level of ROS were investigated with the use of flow cytometry technique. Aria III flow cytometer (Becton Dickinson, Franklin Lakes, United States) with FITC configuration (488 nm excitation; emission: LP mirror 503, BP filter 530/30) or with PE con-figuration (547 nm excitation; emission: 585 nm) was used for the analysis. The cells treated for 5 min with hydrogen peroxide (H_2_O_2_, at doses 3 μM) served as positive control in all cytometric assays. The cells (150,000 cells/mL) were seeded onto 12-well plates and prepared in the same way as for the cytotoxicity assay. Then, the cells were scraped and centrifuged (1,500 rpm, 3 min).

For cell cycle analysis, the cells were suspended in a hypotonic buffer (PBS with 5 mg/mL of citric acid; 1:9 Triton-X solution; RNase 100 μg/mL) containing 100 μg/mL of PI. After incubation for 20 min at room temperature, the samples were transferred to ice and stored until measured.

Apoptosis induction was investigated by means of Annexin-V apoptosis assay (BioLegend, San Diego, United States). Shortly, the cells underwent additional washing with PBS, followed by centrifugation (1,500 rpm, 3 min). The pellet was suspended in cold Annexin-V binding buffer and labeled with FITC-labeled Annexin-V for 30 min at 37 °C in darkness. Next, PI was added (at 100 μg/mL), and the samples were kept on ice until the measurement.

Intracellular ROS content was measured with the use of cell permeable-compound DCFH-DA, which is hydrolyzed inside cells and, due to the presence of ROS, undergoes oxidation, yielding highly fluorescent compound 2′,7′-dichlorofluorescin (DCF). For this experiment, cells were cultivated in the presence of the studied compounds only or with compounds with the addition of an antioxidant, N-acetylcysteine (NAC) at 5 μM. Shortly, harvested cells were washed with PBS and centrifuged (1,500 rpm, 3 min). one more time. Then, DCFH-DA was added at a concentration of 30 μmol/L, and the probes were incubated for 30 min at 37 °C. After incubation, the samples were transferred to ice and stored until measured.

### 2.9 Caspase 3 activity

Caspase 3 activity was measured with the use of a commercially available kit purchased from GenScript Biotech (Leiden, the Netherlands). The cells (150,000 cells/mL) were seeded onto 6-well plates and prepared in the same way as for the cytotoxicity assay. Incubation period with the studied extracts lasted for 48 h (37 °C). Then, the cells were scraped, centrifuged (2,000 rpm, 5 min), and lysed. Caspase 3 activity was monitored by spectrophotometric detection (A_405_) of the chromophore p-nitroanilide (pNA). The ratio of the absorbance of pNA in the studied sample and the untreated sample was used for the calculation of the relative caspase 3 activity.

### 2.10 Intracellular accumulation of rhodamine 123

MDCK and MDCK-MDR1 cells were harvested and incubated (400,000 cells/mL) with the appropriate concentration of the studied compounds (15 min, room temperature). Then, R123 (5 μM) was added, and the cells were incubated for 60 min at 37 °C. After incubation, cells were washed and resuspended in PBS for flow cytometric analysis. Beckman Coulter (Brea, United States) CytoFlex instrument equipped with a 488 nm argon laser and FITC filter (530/30 nm) was applied. A total of 10,000 events were registered and analyzed. Control samples were treated with medium only. The fluorescence intensity ratio (FIR) was calculated from the following equation on the basis of measured fluorescence values (FL).

$$FIR=\frac{\left({FL}_{MDCK-MDR1\;treated}\right)/\left({FL}_{MDCK-MDR1\;control}\right)}{\left({FL}_{MDCK\;treated}\right)/\left({FL}_{MDCK\;control}\right)}$$

### 2.11 Statistical analysis

All the experimental results were expressed as the mean values ±standard deviation (SD) from at least three repetitions. The statistical significance was determined by Student’s t-test (*p < 0.05) with the use of Statistica 10 software.

## 3 Results

### 3.1 UHPLC-MS analysis

**FIGURE 1:**
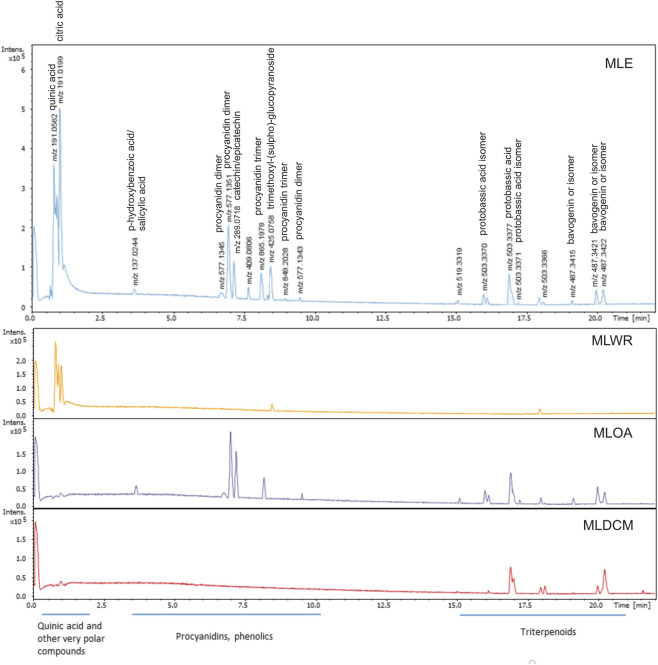
UHPLC-MS chromatograms of the fractions obtained by the partitioning of the aqueous ethanol extract from the bark of *Madhuca longifolia* (MLE). MLWR–water fraction, MLOA–ethyl acetate fraction, and MLDCM–dichloromethane fraction.

The detailed analysis of MLE extract as well as its tentative characterization based on MS spectra was published in our previous work (Środa-Pomianek et al., 2024). Figure 1 presents the chromatograms that illustrate the distribution of compounds present in the MLE extract after its partitioning.

### 3.2 Cell viability

**FIGURE 2:**
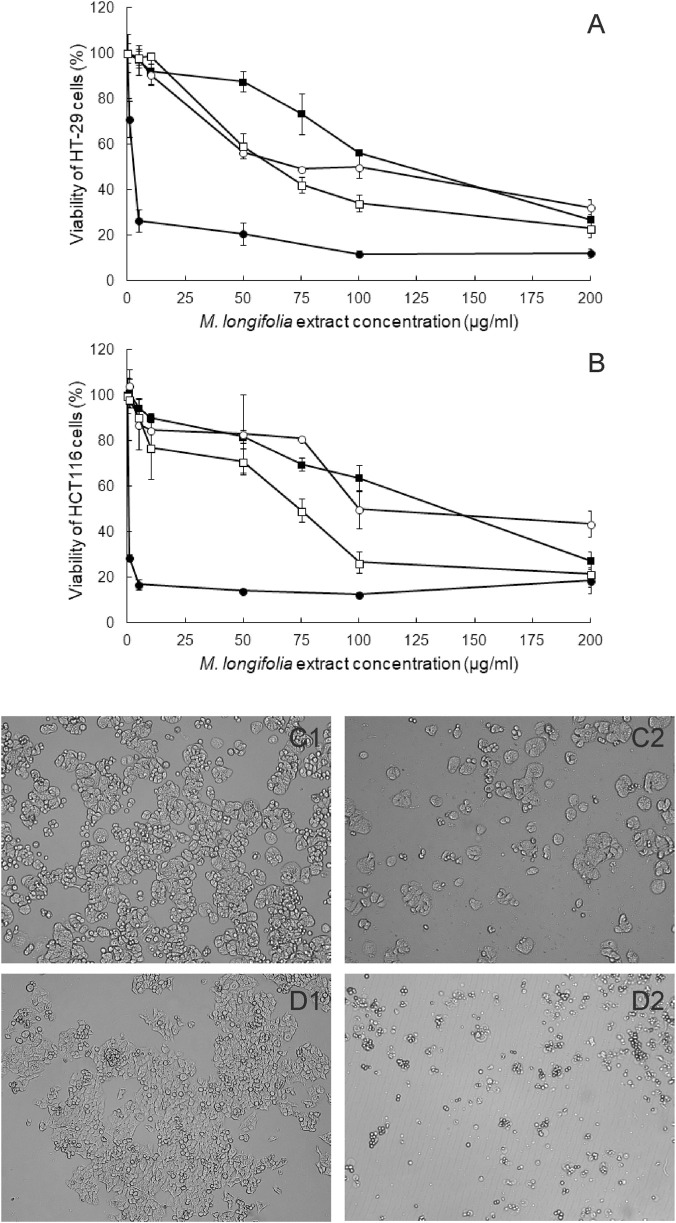
Cytotoxicity of *Madhuca longifolia* extracts in HT-29 **(A)** and HCT116 cells **(B)** determined by SRB assay. MLE–full circles, MLDCM–full squares, MLOA–open circles, MLWR–open squares. Data are presented as means of three experiments ±SD. Microscopic images of untreated HT-29 **(C1)** and HT116 cells **(D1)**, and cells treated by MLE at IC_90_ for 48 h **(C2,D2)**. Magnification was ×10.

Cytotoxicity of the MLE extract from the bark of *M. longifolia* and three fractions (MLDCM, MLOA, and MLWR) towards two different colon cancer cell lines was studied employing the SRB assay. As it can be noticed from the data presented in Figure 2 and Table 1, MLE was an extremely active anticancer agent. Surprisingly, further fractionation of MLE did not result in the enhancement of the cytotoxic activity. All derivative extracts were characterized by much lower ability to inhibit colon cancer cell growth than MLE. In both cell lines, their cytotoxicity increased in the order MLDCM > MLOA > MLWR. Noticeably, HCT116 cells were more vulnerable to the presence of MLE than HT-29 cells. On the other hand, such selectivity between colon cancer lines was not observed for the other studied extracts.

**TABLE 1:** Cytotoxicity of *Madhuca longifolia* extracts towards human colon cancer cells determined by SRB assay.

Extract/Fraction	IC_50_ (µg/mL)	
	HT-29	HCT116
MLE	3.06 ± 0.93	0.012 ± 0.007
MLDCM	174.69 ± 29.54	185.35 ± 36.96
MLOA	84.70 ± 15.51	142.37 ± 11.84
MLWR	67.43 ± 1.07	64.54 ± 11.89

IC_50_ values (concentrations required to reduce cell number by 50%) were calculated with the use of CompuSyn software.

Additionally, the impact of *M. longifolia* extracts on the viability of non-cancer colon epithelial cells was investigated. The number of FHC cells was not reduced by more than 20% by any of the extracts applied at 200 μg/mL.

The viability of cancer cells in the presence of MLE extract and its three fractions was also investigated by means of MTT assay (Supplementary Figure S2). In contrast to the SRB assay, which measures total cellular protein content, the signal in the MTT assay is proportional to the metabolic activity of living cells. As presented in Table 2, both assays yielded similar results. MLE turned out to possess the most potent activity, followed by MLWR, MLOA, and MLDCM, which was the least active.

**TABLE 2:** The effect of *Madhuca longifolia* extracts on the viability of human colon cancer cells determined by MTT assay.

Extract/Fraction	IC_50_ (µg/mL)	
	HT-29	HCT116
MLE	2.13 ± 0.74	0.042 ± 0.017
MLDCM	152.82 ± 21.63	202.54 ± 49.98
MLOA	87.51 ± 8.06	132.15 ± 15.08
MLWR	72.64 ± 15.85	44.16 ± 8.66

IC_50_ values (concentrations required to reduce cell number by 50%) were calculated with the use of CompuSyn software.

### 3.3 Interaction of extracts with anticancer drug

To check whether *M. longifolia* extracts might be potentially useful as adjuncts to anticancer therapy, their anticancer effect was studied in the presence of SN-38. SN-38 is an active form of irinotecan, the inhibitor of topoisomerase I, frequently being a component of chemotherapeutic regiments used to treat colon cancer. For this purpose, cytotoxic activity of the studied extracts was investigated in combination with SN-38, applied in low concentrations, and the analysis of the type of interaction between the drug and the extract was performed. The results are presented in Table 3. For the experiment, mixtures of the extracts and SN-38 were prepared, and their cytotoxicity was compared with the effect of the components applied alone. Each of the extracts was used at a concentration equal to its IC_25_ value, and combined with the drug in various concentrations. Since the great majority of the obtained combination index (CI) values were below 1, it can be stated with full certainty that all the studied extracts interacted synergistically with SN-38. This effect was observed both in HT-29 and HCT116 cell lines. The synergistic effect was more evident for higher concentrations of SN-38. However, it has to be underlined that the concentrations of the drug used in the present experiment were non-toxic (0.002 μg/mL) or only slightly toxic to colon cancer cells (more than 80% of cells stayed viable in the presence of 0.04 μg/mL of the drug).

**TABLE 3:** Combination of *Madhuca longifolia* extracts with SN-38 against colon cancer cell growth.

Cell line	Extract at IC_25_	Concentration of SN-38 (μg/mL)	Ratio	Combination index (CI)
HT-29	MLE	0.002	1 : 205	1.073
		0.020	1 : 20.50	0.521
		0.040	1 : 10.25	0.731
	MLDCM	0.002	1 : 28,185	0.450
		0.020	1 : 2,818.50	0.361
		0.040	1 : 1,409.25	0.457
	MLOA	0.002	1 : 15,880	0.157
		0.020	1 : 1,588	0.070
		0.040	1 : 794	0.209
	MLWR	0.002	1 : 18,795	1.001
		0.020	1 : 1,879.50	0.819
		0.040	1 : 939.75	0.790
HCT116	MLE	0.002	1 : 0.0300	0.942
		0.020	1 : 0.0030	0.700
		0.040	1 : 0.0015	0.638
	MLDCM	0.002	1 : 27,250	0.538
		0.020	1 : 2,725	0.302
		0.040	1 : 1,365.5	0.231
	MLOA	0.002	1 : 30,835	1.240
		0.020	1 : 3,083.50	0.552
		0.040	1 : 1,541.75	0.351
	MLWR	0.002	1 : 20,315	1.153
		0.020	1 : 2,031.50	0.811
		0.040	1 : 1,015.75	0.685

Dose and effect data were obtained from the SRB, assay (mean values of three experiments) and analyzed by CompuSyn software. CI, values were calculated by CompuSyn software. CI, 1 indicates additive effect, CI < 1–synergism, and CI > 1–antagonism.

### 3.4 Analysis of cell cycle

**FIGURE 3:**
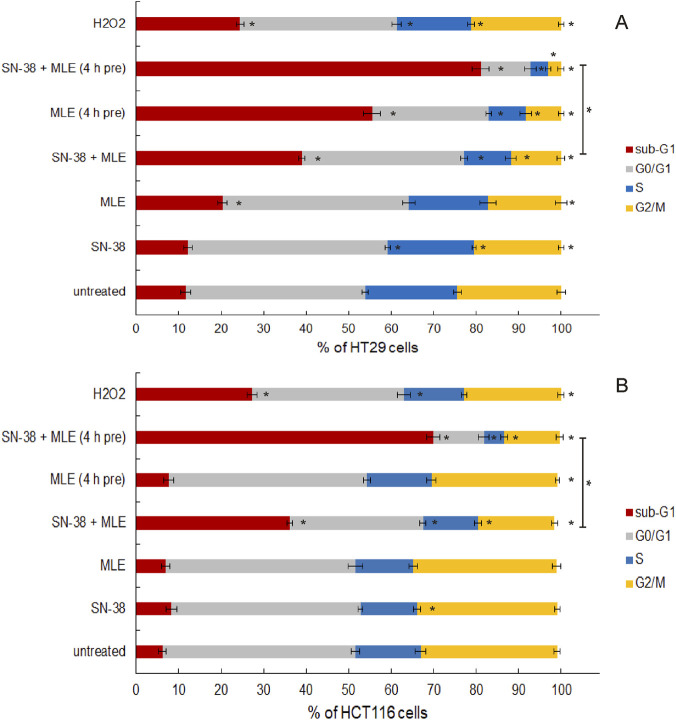
Influence of MLE on the distribution of HT-29 **(A)** and HCT116 cells **(B)** between phases of cell cycle determined on the basis of DNA content. MLE was applied at 0.1 μg/mL and 0.01 μg/mL in HT-29 and HCT116 cells, respectively; SN-38 was applied at 0.002 μg/mL; 4 h pre–pretreatment of cells by MLE for 4 h before the experiment. Sub-G1 population–dead cells, G0/G1 – mononuclear cells, S–DNA replication, G2/M–mitosis. The means of three experiments ±SD are presented (*p < 0.05).

**FIGURE 4:**
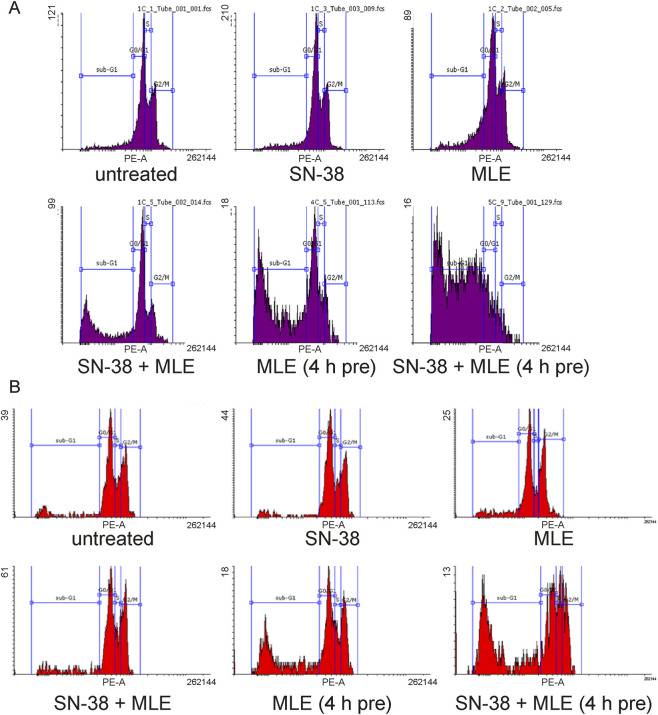
Exemplary histograms of DNA content in HT-29 **(A)** and HCT16 cells **(B)** treated with MLE (at 0.1 μg/mL in HT-29 and 0.01 μg/mL in HCT116 cells), SN-38 (at 0.002 μg/mL), and their combination. 4 h pre–pretreatment of cells by MLE for 4 h before the experiment.

As MLE turned out to be the extract of the most potent anticancer activity, further studies were limited to this substance. To explore the character of the interaction of MLE with the anticancer drug in detail, the influence of low concentrations of both substances and their mixture on the cell cycle distribution of colon cancer cell was investigated. The affiliation of cells to the appropriate phase of the cycle was determined on the basis of their DNA content, analyzed by flow cytometry. As can be seen in Figures 3, 4, in both HT-29 and HCT116 cells, SN-38 at 0.002 μg/mL did not influence the sizes of the population of cells in various cell cycle phases. Similarly, MLE at 0.1 μg/mL did not influence HT-29 cell cycle distribution and MLE at 0.01 μg/mL did not significantly affect HCT116 cells. When the mixture of MLE and the drug was applied the number of dead (fragmented) cells was significantly increased, and the number of cells being alive, undergoing replication and mitosis was reduced. A similar, but even stronger, effect was observed when colon cancer cells were pre-treated with MLE for 4 h before adding SN-38. Such an effect was observed in both HT-29 and HCT116 cells. There was, however, one difference between the cell lines. Namely, when the cells were pretreated by MLE alone, no influence of the extract was noticed in HCT116 cells, while in HT-29 cells a potent increase in the number of dead cells was recorded.

### 3.5 Induction of apoptosis

**FIGURE 5:**
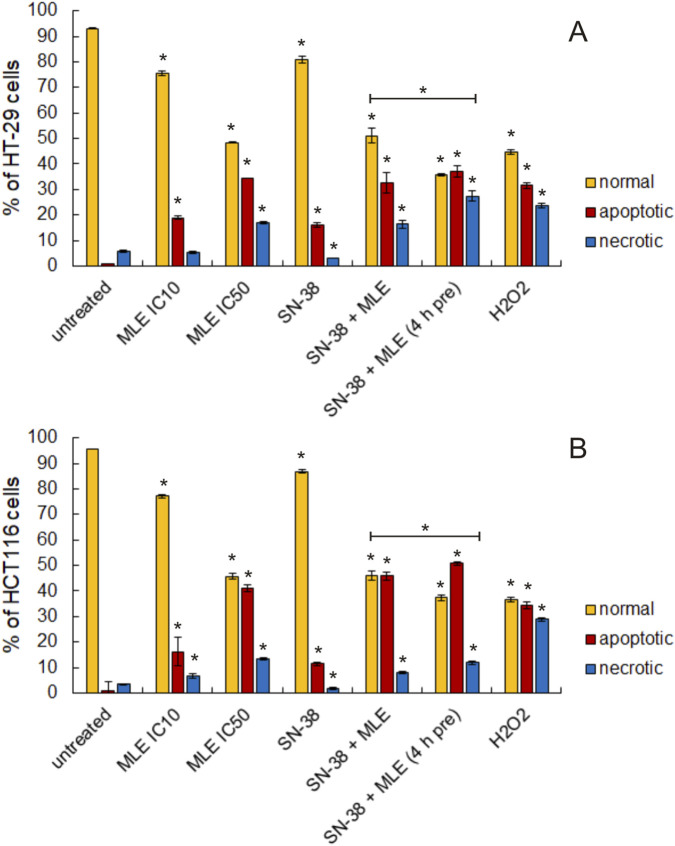
Influence of MLE (at its IC_10_ and IC_50_), SN-38 (at 0.002 μg/mL), and their mixture (MLE at IC_10_ and SN-38 at 0.002 μg/mL) on the proportion of normal, apoptotic and necrotic cell populations in HT-29 **(A)** and HCT116 cells **(B)** as determined by Annexin-V assay. 4 h pre–pretreatment of cells by MLE for 4 h before the experiment. The means of three experiments ±SD are presented (*p < 0.05).

**FIGURE 6:**
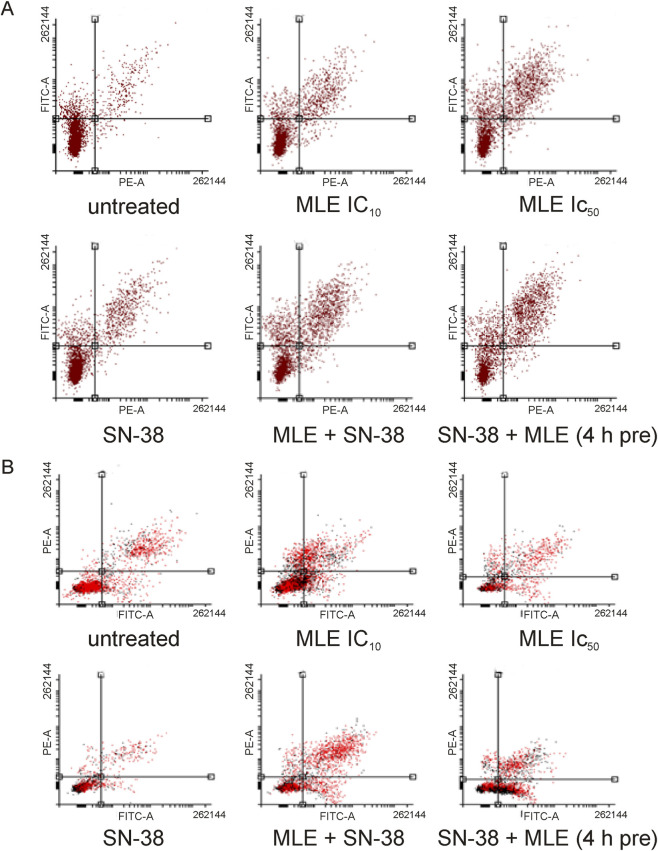
Exemplary histograms of recorded in HT-29 **(A)** and HCT116 cells **(B)** cells stained with Annexin-V and PI. Cells were treated with MLE (at IC_10_ and IC_50_), SN-38 (at 0.002 μg/mL), and their mixture (MLE at IC_10_ and SN-38 at 0.002 μg/mL). 4 h pre–pretreatment of cells by MLE for 4 h before the experiment. 0.1 μg/mL in HT-29 and 0.01 μg/mL in HCT116 cells), SN-38 (at 0.002 μg/mL), and their combination. 4 h pre–pretreatment of cells by MLE for 4 h before the experiment. Influence of MLE at different concentrations (expressed in μg/mL) on relative caspase three activity in HT-29 **(A)** and HCT116 cells **(B)**. SN-38 was applied at 0.002 μg/mL; positive control camptothecin at 10 μmol/L. MLE at concentration of 0.1 μg/mL was used in the mixtures with SN-38. The means of three experiments ±SD are presented (*p < 0.05).

Next, the ability of MLE and MLE combined with SN-38 to induce apoptosis was studied. For this purpose, a flow cytometric assay based on the detection of phosphatidylserine in the outer monolayer of cellular membrane was applied. In both cell lines MLE was demonstrated to significantly increase the number of apoptotic cells in a concentration-dependent manner (Figures 5, 6). SN-38 at 0.002 μg/mL concentration also increased this parameter, however the effect was moderate. On the other hand, a strong increase in the size of the apoptotic cell population was detected when MLE and SN-38 were applied in combination. This effect was also significantly intensified when colon cancer cells were pretreated with the extract before the addition of the anticancer drug.

**FIGURE 7:**
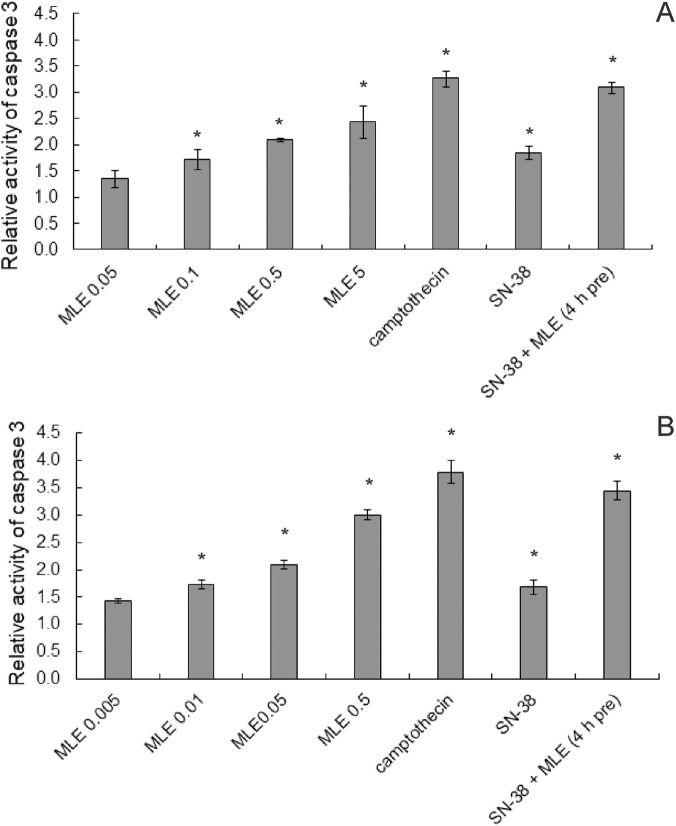
Influence of MLE at different concentrations (expressed in μg/mL) on relative caspase three activity in HT-29 **(A)** and HCT116 cells **(B)**. SN-38 was applied at 0.002 μg/mL; positive control camptothecin at 10 μmol/L. MLE at concentration of 0.1 μg/mL was used in the mixtures with SN-38. The means of three experiments ±SD are presented (*p < 0.05).

Additionally, the influence of the studied substances on the activity of caspase 3, an essential enzyme of the executive phase of apoptosis, was investigated. As presented in Figure 7, MLE caused a significant increase in the activity of this proteolytic enzyme in both HT-29 and HCT116 cells, and the effect was concentration-dependent. SN-38 alone also had an effect, causing a c. a. 1.5-fold raise in caspase 3 activity. When HT-29 cells were pretreated with 0.1 μg/mL of MLE for 4 h before adding SN-38, the activity of caspase 3 was *c. a.* 3 times higher than under control conditions. The same experiment performed in HCT116 cells yielded the result of a 3.5-fold increase of enzyme activity.

### 3.6 Reactive oxygen species level

**FIGURE 8:**
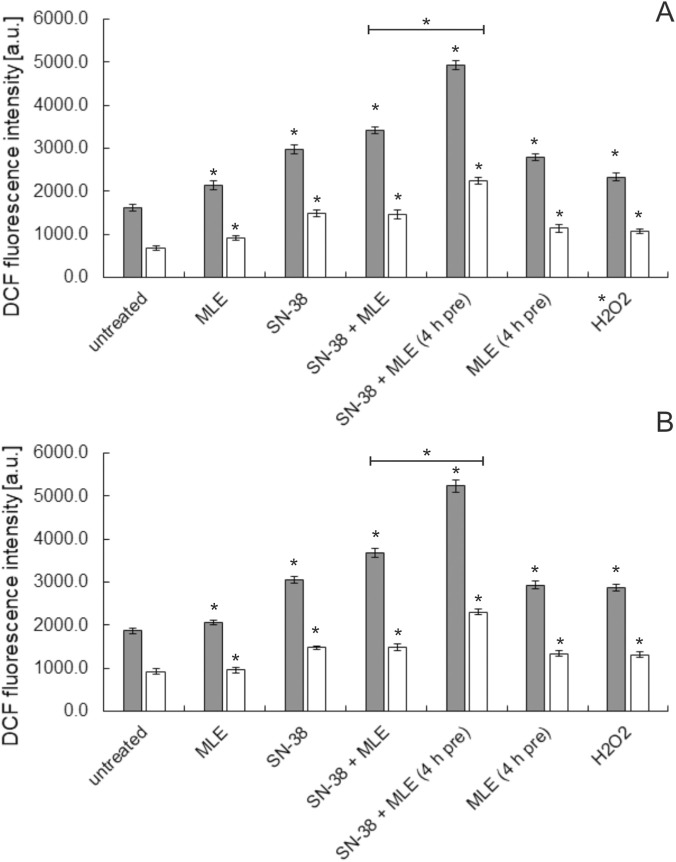
Influence of MLE (at 0.1 μg/mL in HT-29 cells and 0.01 μg/mL in HCT116) on ROS level in HT-29 **(A)** and HCT116 cells **(B)**. SN-38 was applied at 0.002 μg/mL; 4 h pre–pretreatment of cells by MLE for 4 h before the experiment. The means of three experiments ±SD are presented (*p < 0.05).

Since cytotoxic activity is often associated with the ability of chemical compounds to induce the production of ROS in cells, the prooxidative properties of *M. longifolia* ethanolic extract alone and in combination with SN-38 were also investigated. The results shown in Figure 8 demonstrated that MLE slightly increased cellular ROS levels in both colon cancer cell lines, and the effect was potentiated when the time of incubation with the extract was prolonged. However, SN-38 in the concentration that is virtually non-toxic to the studied cells turned out to be stronger prooxidative agent than MLE. When both substances were applied together, a further increase in ROS level was observed, and the effect was potentiated when colon cancer cells were pretreated with the extract before the addition of SN-38.

Additionally, the experiments with the use of an antioxidant N-acetylcysteine (NAC) were performed (Figure 8). As supposed, the presence of NAC reduced the amount of ROS produced by colon cancer cells. However, the potentiation of ROS production in the presence of MLE and SN-28 applied together was still present, especially when the cells were pretreated with MLE.

### 3.7 Interaction with ABCB1 transporter

One of the potential mechanisms of the observed potentiation of SN-38 activity by MLE could be the inhibition of multidrug transporter, ABCB1 (P-glycoprotein). To check this possibility, a rhodamine 123 uptake test was performed. MDCK cells and MDCK-MDR1 cells, which are transfected with the human *ABCB1* gene, were used as a model system. Rhodamine 123 is an ABCB1 substrate and, following its intracellular uptake, allows for the estimation of the transporter activity. The results are presented in Table 4. As can be noticed, neither SN-38 nor MLE inhibited ABCB1 in concentrations in which their synergistic anticancer activity was observed. MLE slightly inhibited the transport activity of ABCB1 only in concentrations above 5 μg/mL. It was therefore not likely that inhibition of the multispecific drug transporter was responsible for the observed effect of enhancement of SN-38 activity by MLE.

**TABLE 4:** Intracellular accumulation of R123 in MDCK-MDR1 cells treated with MLE, SN-38, and both simultaneously. ABCB1 inhibitor, verapamil, was used as a positive control. Means ± SD of three presented.

Compound	Concentration (μg/mL)	FIR
MLE	1.0	0.98 ± 0.17
	2.5	1.01 ± 0.14
	5.0	1.02 ± 0.19
	7.5	1.43 ± 0.22
	10	1.12 ± 0.19
SN-38	0.002	0.91 ± 0.21
	0.010	0.95 ± 0.21
	0.020	0.96 ± 0.17
MLE + SN-38	0.1 + 0.002	1.04 ± 0.15
Verapamil	11.3	2.49 ± 0.29

## 4 Discussion

Aqueous ethanolic extract from the bark of *M. longifolia* turned out to be a very potent anticancer agent able to inhibit the growth of colon cancer cells. On the other hand, MLE did not inhibit the growth of non-cancerous colon epithelial cells significantly. These results point to some degree of selectivity of MLE, which was more cytotoxic to cancerous than to normal cells. The HCT116 cell line was especially sensitive to the extract, and the IC_50_ value of MLE recorded was in the nanogram/mL range. MLE was also cytotoxic towards HT-29 cells, however its concentration required to kill 50% of cells lay in the microgram/mL range. The difference in sensitivity to the extract between the two colon cancer cell lines was quite striking. This was most probably caused by the dissimilar characteristics of the cell lines. Although named collectively colon cancer, various cell lines used as laboratory model systems show different molecular features. When the status of mutations in colon cancer cell lines was investigated, it was demonstrated that only KRAS (a proto-oncogene in the RAS/RAF/MAPK pathway) and PIK3CA genes (leading to hyperactivation of the PI3K/AKT pathway) were mutated in HCT116 cell line (Ahmed et al., 2013). On the other hand, HT-29 cells beared mutations in proto-oncogene BRAF, PIK3CA, TP53 (pivotal in maintaining genome integrity), and SMAD4 (a signal transducer of TGFβ pathway) (Ahmed et al., 2013; Voutsadakis, 2022). Additionally, HCT116 was a highly aggressive cell line, but its capacity to differentiate was limited, whereas HT-29 cells possessed an intermediate capacity to differentiate into enterocytes and mucin-expressing cells (Yeung et al., 2010). This short analysis allowed us to conclude that HT-29 cells seemed more distant from wild-type colon epithelium cells as compared to HCT116 cells. This could elucidate the higher sensitivity of HCT116 cells to the treatment.

Previously, we have studied the activity of the same *M. longifolia* extract in human melanoma cells (Środa-Pomianek et al., 2024). In this type of cancer cells, MLE was also a potent antiproliferative agent with IC_50_ values ranging from 2.5 to 15 μg/mL depending on the type of cell line.

In hope of identifying the compounds responsible for the anticancer activity of MLE, the extract was further fractioned. Unfortunately, the cytotoxicity of the three derivative fractions towards colon cancer cells was much lower than the activity of the native MLE extract. In both cancer cell lines, MLWR exhibited the highest activity followed by MLOA and MLDCM. Although disappointing, this observation did not come as a surprise. Extracts obtained from medicinal plants may contain thousands of individual chemical compounds in various proportions and quantities (Enke and Nagels, 2011). Therefore, the biological activity of the extracts is likely to result from the combined action of their multiple components, and their interactions might be additive, synergistic or antagonistic (Wagner and Ulrich-Merzenich, 2009; Rasoanaivo et al., 2011). Synergy may occur due to the enhancement of bioavailability, modulation of metabolism of one component by the other, or by their complementary mechanism of action (Rasoanaivo et al., 2011). The examples might be the increase of absorption of curcumin by piperine (Balakumar et al., 2022), their synergistic anticancer activity (Cho et al., 2024), and the dissimilar influence of turmeric and black and red pepper on absorption of carotenoids by Caco-2 cells (Shilpa et al., 2021). That is why the isolation of active chemicals from active extracts is not always possible, and plant extracts frequently exhibit better biological activity than isolated compounds. Synergistic effect are also the reason for which traditional healing approaches, like Chinese or Ayurvedic medicine, typically use the combination of several plants in traditional recipes.

Additionally, aqueous ethanol extract of *M. longifolia* and its fractions were investigated for their interaction with SN-38, a drug commonly used in the treatment of colon cancer. As a result of analysis with the use of CompuSyn software, it was found that all the entities interacted synergistically with the anticancer drug in both colon cancer cell lines. Previously, synergy between MLE and dacarbazine in human melanoma cells was demonstrated (Środa-Pomianek et al., 2024). Also, other plant extracts have been shown to enhance the action of anticancer drugs. For example, Asian ginseng extracts as well as extracts from needles and twigs of *Taxus cuspidata* exhibited a synergistic effect in combination with 5-fluorouracil (Fishbein et al., 2009; Shang et al., 2011), both extracts from *Rauwolfia vomitoria* and from *Clinacanthus nutans* potentiated the activity of gemcitabine (Yu and Chen, 2014; Hii et al., 2019), while red beetroot extract and *Caesalpinia spinosa* extract formed synergistic pairs with doxorubicin (Kapadia et al., 2013; Sandoval et al., 2016).

To further elucidate the anticancer activity of MLE, its ability to induce apoptosis was tested. It was discovered that the extract significantly increased the number of apoptotic cells in colon cancer cells. Additionally, the extract increased the activity of apoptosis associated enzyme, caspase 3, in a concentration dependent manner in both HT-29 and HCT116 cells. From the obtained results, it was therefore clear that MLE possessed proapoptotic activity, especially in higher concentrations. Similar results were previously obtained for MLE in human melanoma cells (Środa-Pomianek et al., 2024).

It was also demonstrated that the MLE applied in low concentrations was able to increase the intracellular ROS level in colon cancer cells. It was in agreement with our previous study on this extract in human melanoma cells (Środa-Pomianek et al., 2024). However, some discrepancy existed with some former works in which extracts from different parts of *M. longifolia* were shown to possess antioxidative activity (Jose et al., 2018; Dubey et al., 2019). However, these studies utilized simple model systems such as DPPH or lipid peroxidation assays, while whole cells were used in the present work. In our opinion, this was likely to be the cause of the apparent inconsistency, especially as the increase in ROS generation treated with the extract from *M. longifolia* leaves was also reported in breast cancer cells (Sarkar et al., 2018).

The effect of MLE combined with SN-38 was of particular interest since the assessment of MLE as a putative adjunct to colon cancer chemotherapy was one of the goals of the present work. In the experiments, a non-toxic concentration (0.002 μg/mL) of anticancer drug was used, and MLE was applied either at the concentration of 0.1 μg/mL in HT-29 cells or 0.01 μg/mL in HTC116 or at its IC_10_, depending on the experiment. It was found that MLE in such concentrations had no influence on the cell cycle of colon cancer cells. However, the extract, even in low concentrations, increased the size of the apoptotic cell population, activated caspase-3, and increased the production of ROS in both HT-29 and HCT116 cells. Activity of SN-29 applied alone (at 0.002 μg/mL) was similar and comparable in magnitude to the activity of MLE. When the two substances were used in combination, significant potentiation of all types of activities was observed as compared to both substances alone. Moreover, the pretreatment of colon cancer cells with MLE for 4 h before the application of SN-38 led to an even further increase in the activity of the mixture in all studied aspects. The experiments on ROS generation performed in the presence of an antioxidant NAC showed the reduction of the effect of MLE on SN-38 activity but NAC did not cancelled the effect of potentiation of activity when both SN-38 and MLE were applied simultaneously. It was noticed that the pretreatment with MLE in some way made colon cancer cells more sensitive to the action of the chemotherapeutic drug, as if MLE sensitized the cells to the drug. Similar observations were also made in the case of MLE applied to melanoma cells in combination with dacarbazine (Środa-Pomianek et al., 2024]. The mechanism of the recorded effect is currently unknown. However, it was noticed that the prolonged treatment of colon cancer cells with MLE (4 h + 48 h) resulted in increased ROS production and, in the case of HT-29 cells only, also an increased number of cells in sub-G1 phase (dead, fragmented). In their work on colon and lung cancer cells, Riganti et al. (2015) observed that two low doses of doxorubicin applied in a 24-h interval were more toxic to the cells than a single dose 5 times higher. It was explained by the more potent increase in cellular ROS level induced by the first protocol. This situation is somehow similar to the effect noticed in the present work, so it was likely that the ROS-associated mechanism might also be responsible for the observed increased activity of the MLE-drug mixture when the cells had been pretreated *by M. longifoli*a extract.

In search of the potential mechanism of the observed synergy between the anticancer drug and *M. longifolia* extract, their activity on the multispecific transporter, ABCB1, was investigated. The transporter is commonly engaged in cancer cells’ multidrug resistance by reduction of the drugs’ uptake and reducing their intracellular concentration below the killing threshold. However, it was found that the effect of both MLE and SN-38 on the transport activity of ABCB1 was negligible. So the interaction of the studied substances with this cellular transport system was unlikely to be responsible for the observed effects.

To summarize, MLE itself demonstrated strong anticancer activity in the studied colon cancer cell lines. Unfortunately, at present, it has not been successful to identify fractions and chemical compounds that were directly responsible for its activity. It is probable that the synergism among many chemical entities present in MLE all is the key factor for the overall promising activity. On the other hand, future experiments on identifying the most biologically important MLE components are highly desirable. Moreover, in the present work, the synergy between the studied extract and SN-38 was demonstrated. Future studies are needed to fully elucidate the mechanism of the observed interaction. Among others, the effect on MLE on SN-28 uptake or efflux via different cellular transport systems (transporter inhibition and/or induction), its direct effect on topoisomerase,I or its ability to induce various types of cell death should be studied.

## 5 Conclusion

In conclusion, aqueous ethanol extract of *M. longifolia* bark was shown to possess significant anticancer properties in colon cancer cells. The ability to induce apoptosis and ROS production within the cells might contribute to the MLE-induced cell death. Some degree of selectivity was also observed since the extract was more toxic towards cancer cells than towards normal colon epithelium. What was more important, the combination of MLE with anticancer drug SN-38 turned out to be very active against colon cancer cells, and the existence of synergy between both substances was demonstrated. Taken together, the results obtained in the present work pointed to the investigated extract from *M. longifolia* bark as a source of promising adjunct substances that could be potentially used in therapy in order to decrease the concentrations of irinotecan applied to the patients and, in this way, to reduce the side-effects of the drug.

## Data Availability

The raw data supporting the conclusions of this article will be made available by the authors, without undue reservation.
