# Supplementary material for: Madhuca longifolia bark extract as an adjunct to the treatment of colon carcinoma cells with SN-38: a synergistic combination with potent activity
Source: Front Pharmacol. 2026 May 12;17:1804862. doi: 10.3389/fphar.2026.1804862 (PMC13201237; doi:10.3389/fphar.2026.1804862)
Supplement: Supplementary file 1 [file Supplementaryfile1.docx]

**Figure S1.** Cytotoxicity of SDS in HT-29 (full circles) and HCT116 cells (open circles) determined by SRB assay. Data are presented as means of three experiments ± SD.

**Figure S2.** Viability of HT-29 (**A**) and HCT116 cells (**B**) determined by MTT assay. MLE – full circles, MLDCM – full squares, MLOA – open circles, MLWR – open squares. Data are presented as means of three experiments ± SD.

**A**

**B**

**Figure S3.** Cytotoxicity of SDS in HT-29 (full circles) and HCT116 cells (open circles) determined by MTT assay. Data are presented as means of three experiments ± SD.
